# A Machine‐Learning‐Based Bibliometric Analysis of Cell Membrane‐Coated Nanoparticles in Biomedical Applications over the Past Eleven Years

**DOI:** 10.1002/gch2.202200206

**Published:** 2023-02-22

**Authors:** Yiyin Zhang, Shengxi Jin, Duguang Li, Guoqiao Chen, Yongle Chen, Qiming Xia, Qijiang Mao, Yiling Li, Jing Yang, Xiaoxiao Fan, Hui Lin

**Affiliations:** ^1^ Department of General Surgery Sir Run Run Shaw Hospital School of Medicine Zhejiang University Hangzhou 310016 P. R. China; ^2^ Zhejiang Engineering Research Center of Cognitive Healthcare Sir Run Run Shaw Hospital School of Medicine Zhejiang University Hangzhou 310016 P. R. China

**Keywords:** bibliometrics, cell membrane encapsulation, medical applications, nanoparticles

## Abstract

Cell membrane encapsulation is a growing concept in nanomedicine, for it achieves the purpose of camouﬂage nanoparticles, realizing the convenience for drug delivery, bio‐imaging, and detoxification. Cell membranes are constructed by bilayer lipid phospholipid layers, which have unique properties in cellular uptake mechanism, targeting ability, immunomodulation, and regeneration. Current medical applications of cell membranes include cancers, inflammations, regenerations, and so on. In this article, a general bibliometric overview is conducted of cell membrane‐coated nanoparticles covering 11 years of evolution in order to provide researchers in the field with a comprehensive view of the relevant achievements and trends. The authors analyze the data from Web of Science Core Collection database, and extract the annual publications and citations, most productive countries/regions, most influential scholars, the collaborations of journals and institutions. The authors also divided cell membranes into several subgroups to further understand the application of different cell membranes in medical scenarios. This study summarizes the current research overview in cell membrane‐coated nanoparticles and intuitively provides a direction for future research.

## Graphical abstract

This article presents a literature analysis of cell membrane‐coated nanoparticles covering 11 years of evolution based on the data from the Web of Science Core Collection, including the annual publications and citations, most productive countries/regions, most influential scholars, the collaborations of journals and institutions. It gives a current research overview in this field and intuitively provides a vision for researchers.

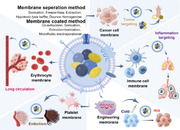

## 1 Introduction

Stability and targeted drug delivery are urgent problems for nanoparticles in medical applications. The traditional solution is to add polymer materials on the surface of nanoparticles but hard to avoid immune system recognition.^[^ ^1^ ^]^ Cell membrane encapsulation helps to protect nanoparticles from immune system attack and enhanced targeted ability. Cell membrane biomimetic nanomedicine was first brought up by Zhang et al. in 2011 by coating nanoparticles with an erythrocyte membrane.^[^ ^2^ ^]^ The nanoparticle's circulation time increased to 40 h in vivo, greatly extending the half‐life period, and the red blood cell membrane (RBCM)‐encapsulated nanoparticles can be used multiple times without irritating an immune response. Since then, cancer cell membranes (CM) were applied to design anticancer vaccination and drug delivery in 2014;^[^ ^3^ ^]^ platelet membranes (PM) were applied to escape macrophage phagocytosis in 2015;^[^ ^4^ ^]^ leukocyte membranes as one of the immune cell membranes (IM) were purified to avoid immune system clearance in 2013;^[^ ^5^ ^]^ T‐cell membranes were exploited to target tumor, using their distinct targeting characteristics in 2017,^[^ ^6^, ^7^ ^]^ and other cell membranes‐coated research flourished like bamboo shoots after a spring rain.^[^ ^8^, ^9^ ^]^

Bibliometric analysis helps scholars to gain an overall view of a complex field quickly including acknowledgment of the main publishing institutions, the most influential scholars, and the most representative research. It was able to assist with developing approaches focused on prognosticating the current and future directions of certain areas. It also structured in a more newfangled display mode, compared with systemic reviews which mainly aim at the content of research produced in a certain field.^[^ ^10^ ^]^ It offers a global perspective, which can more comprehensively evaluate the phylogeny of a certain field, and provides a basis for the prediction of future research hotspots.^[^ ^11^ ^]^

**Scheme 1:**
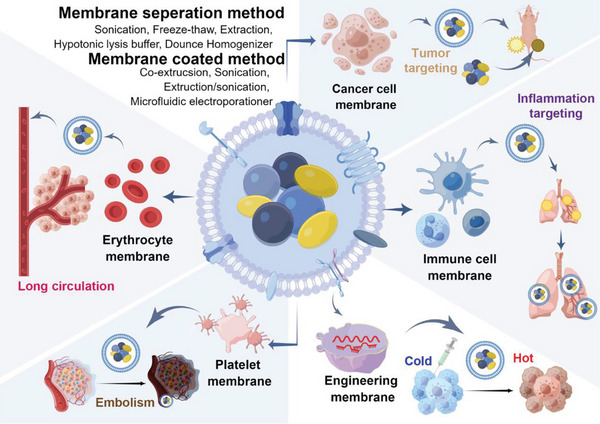
The preparation methods of cell membranes and categories of cell membranes in different medical applications. In the upper left corner, the preparation methods of cell membranes included the membrane separation method and membrane coated method. View in counterclockwise direction, erythrocyte membranes were able to prolong the circulation time of nanoparticles; platelet membranes could help embolize the new vessels derived from tumors; cancer cell membranes exerted their tumor‐targeting ability; immune cell membranes had the potential to target the inflammatory sites; engineering cell membrane became a new research direction, which could improve the immunosuppression in the tumor microenvironment to become immune activated. Drawn with assistance from Figdraw.

Many original articles have been published from 2011 to 2021 in the field of cell membrane‐encapsulated nanoparticles research. In this article, we provided an overview and scientific analysis of research about cell membrane‐encapsulated nanoparticles via data collected from the Web of Science Core Collection (WoSCC). The analyzed indexes include annual publications and citations, most productive countries, most influential and high‐cited authors, and collaborated journals, as well as institutions. These were demonstrated and visualized by several bibliometric software. Moreover, the profiles of different membranes were analyzed independently, including RBCM, PM, CM, IM, and other cell membranes, to understand the different applications in this field by comparing the publications of these five types of cell membranes (**Scheme** **1**). This study revisits the development history of different cell membrane‐encapsulated nanomaterials. It further discusses the application popularity of different cell membranes and the disease scenarios suitable for medical applications.

## 2 Results

### 2.1 The Overview of Global Productions

**Figure 1:**
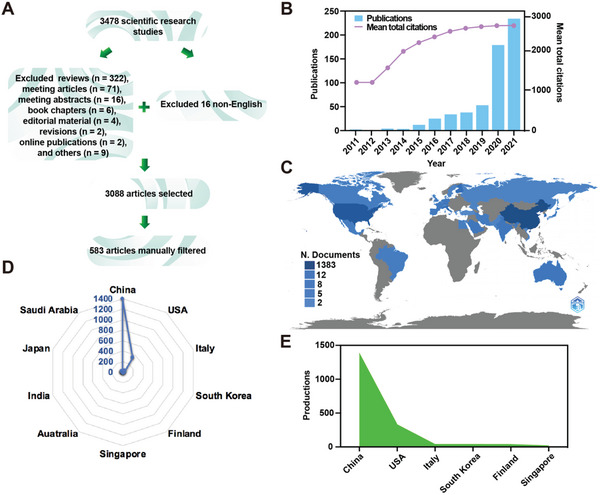
Overall distribution of publications and productions in the cell membrane‐coated nanoparticles field. A) Flowchart of the publications screening process. B) Global annual output and total citation trends. C) Geographical distribution of global production in this field; the according radar map is presented in (D). E) The top six countries of production in this field.

Five hundred and eighty‐three articles associated with cell membrane‐coated nanoparticles were screened out from the database of WoSCC. These documents have been cited 2644 times until December 31st, 2021, with a mean of 11.3 times per publication. The flowchart of the inclusion criteria is demonstrated in **Figure** **1**A. The annual number of publications and citations are shown in Figure 1B. The growth speed of total publications accelerated since 2020 (*n* = 179) compared with that in 2011 to 2019 (*n* = 170), indicating the potential of cell membrane‐coated nanoparticles was gradually accepted by researchers in biomedicine. The mean total citation rate has increased steadily over the years. As shown from the global productivity in Figure 1C, the China and USA are the top two most productive countries worldwide. The top ten most productive countries are exhibited in Figure 1D and **Table** **1**, with 1383 publications in China, 330 publications in the USA, 39 publications in Italy, 39 publications in South Korea, 39 publications in Finland, and 18 publications in Singapore (Figure 1E). The annual publications and citations suggested that there was an increased trend in the development of cell membrane‐coated nanoparticles.

**Table 1:** The top ten productive countries concerning cell membrane‐coated nanoparticles

Rank	Country	Frequencies	Total citations	Average article citations
1	China	1383	15 530	34.5
2	USA	330	9094	121.3
3	Italy	39	48	16
4	South Korea	39	169	18.8
5	Finland	37	99	14.1
6	Singapore	18	77	38.5
7	Australia	12	92	18.4
8	France	12	112	18.7
9	Japan	12	146	48.7
10	Saudi Arabia	12	107	35.7

### 2.2 Collaborations among Countries

**Figure 2:**
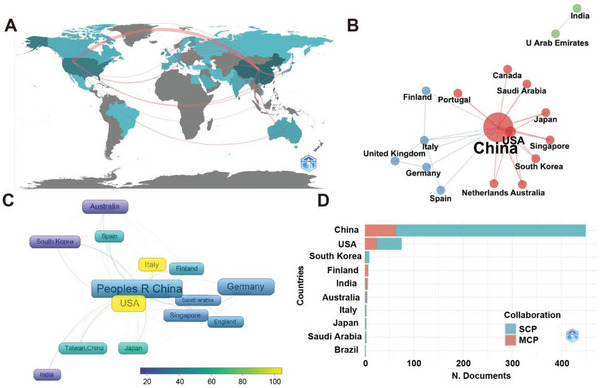
Summaries of countries with close cooperation in the cell membrane‐coated nanoparticles field. A) Geographical distribution of cooperation among countries globally. B) Network visualization cluster map of research collaboration among countries in this field (the node sizes represented the number of productions). C) The most contributed countries with close cooperation in the cell membrane‐coated nanoparticles field. D) International collaboration in cell membrane‐coated nanoparticles research among authors (SCP: single country production; MCP: multiple countries production).

From 2011 to 2021, a total of 31 countries were included in the analysis of cell membrane‐coated nanoparticles. First, we conducted the collaborative connections among countries in **Figure** **2**A and found that countries around the world closely collaborated in this field, especially between China and the USA. We further elaborated on the academic cooperative network between countries by R bibliometrix package (Figure 2B), three clusters were algorithmically generated; the cooperation was closer in the red cluster, for the connections between the nodes were thicker; the cooperation was less in the blue and green clusters, for the connections between the nodes are thinner. The USA mostly collaborated with Asian countries, while China collaborated with both Asian countries and European countries, such as Italy, Portugal, and Germany. The top five countries with the most scientific production were China, the USA, Italy, South Korea, and Finland (Figure 2C). As for the single country publication (SCP), China, the USA, and South Korea were the top three countries with frequent cooperation among corresponding authors; as for the multiple country publication (MCP), China, the USA, and Finland were the top three most cooperative countries according to the affiliates of corresponding authors (Figure 2D).

### 2.3 Most Influential Authors and Cooperation Network

**Figure 3:**
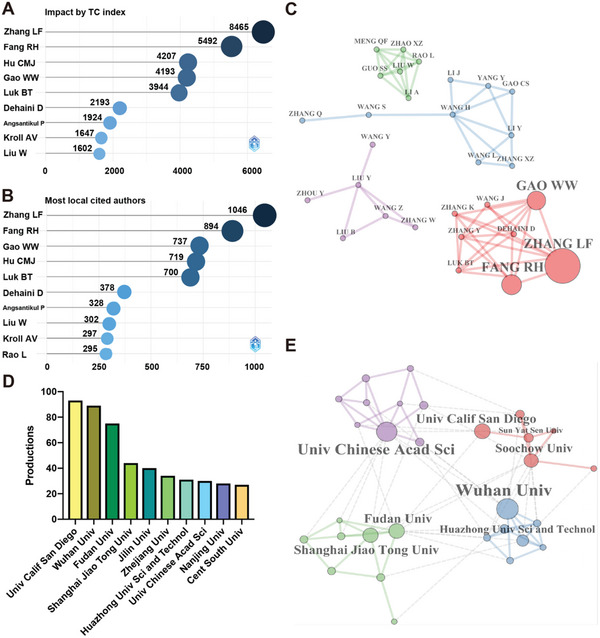
Illustrations of most influential authors and collaborative network among authors and institutions. A) The authors with the most high‐cited total citation index in the cell membrane‐coated nanoparticles field; and the most locally cited authors are shown in (B). C) Four collaboration clusters were generated among authors; the node sizes were based on the number of publications. D) The top ten most productive institutions in the cell membrane‐coated nanoparticles field; the network visualization of their cooperation is shown in (E).

A total number of 2915 authors have devoted to cell membrane‐coated nanoparticles over the past 11 years, and the most productive authors are listed in **Table** **2**. Among the high‐impact authors presented in **Figure** **3**A, Liangfang Zhang, from the University of California San Diego, was the most influential author with a total of 8465 citations. Then it was followed by Ronnie H Fang (*n* = 5492), Che‐Ming J Hu (*n* = 4207), Weiwei Gao (n = 4193), Brian T Luk (*n* = 3944), Diana Dehaini (*n* = 2193), Pavimol Angsantikul (*n* = 1924), Ashley V Kroll (*n* = 1647), and Wei Liu (*n* = 1602). Local citations measure the times an author (or a document) included in this collection has been cited by the documents also included in the collection. Liangfang Zhang was also the most locally cited author (*n* = 1046), followed by Ronnie H Fang (*n* = 894), Weiwei Gao (*n* = 737), Che‐Ming J Hu (*n* = 719), Brian T Luk (*n* = 700), Diana Dehaini (*n* = 378), Pavimol Angsantikul (*n* = 328), Wei Liu (*n* = 302), Ashley V Kroll (*n* = 297), and Lang Rao (*n* = 295, Figure 3B). Ronnie H Fang, Weiwei Gao, Che‐Ming J Hu, Diana Dehaini, Pavimol Angsantikul, and Ashley V Kroll were all scholars from the department of NanoEngineering, the University of California San Diego, who worked with Liangfang Zhang.

**Table 2:** The most productive authors concerning cell membrane‐coated nanoparticles

Author	Country	h_index	g_index	m_index	TC^a)^	NP^b)^	PY_start^c)^
Liangfang Zhang	USA	27	39	2.25	8465	51	2011
Ronnie H Fang	USA	20	28	1.67	5492	28	2011
Che‐Ming J Hu	USA	10	10	0.83	4207	10	2011
Weiwei Gao	USA	21	28	2.10	4193	28	2013
Brian T Luk	USA	12	12	1.20	3944	12	2013
Diana Dehaini	USA	9	10	0.90	2193	10	2013
Pavimol Angsantikul	USA	7	7	0.88	1924	7	2015
Ashley V Kroll	USA	5	6	0.63	1647	6	2015
Wei Liu	China	15	17	1.88	1602	17	2015

^a)^ TC = Total citations

^b)^ NP = the total number of papers published

^c)^ PY = Publication year.

We could analyze the authors’ cooperation from Figure 3C, Liangfang Zhang, Ronnie H Fang, and Weiwei Gao had a closer collaboration with others. Their interest in cell membrane biomimetic nanomedicine developed rapidly and multiplied with Brian T Luk, Diana Dehaini, Joseph Wang, Kang Zhang, and other scholars. However, some scholars were scattered in different domains, for example, Yang Liu independently cooperated with Yun Zhou, Zhou Wang, Bin Liu, Yin Wang, and Wei Zhang. Therefore, we believed that the cooperation between scholars could be further strengthened to achieve a kind of contention and blooming of a hundred schools of thought. During this decade, there were 491 publications contributed to this field from the top ten academic institutes, and the number of publications and percentage of each institution are listed in **Table** **3**.

**Table 3:** The top ten productive institutes concerning cell membrane‐coated nanoparticles

Affiliations	Country	Articles	Counts [%]
University of California San Diego	USA	93	4.72
Wuhan University	China	89	4.52
Fudan University	China	75	3.81
Shanghai Jiao Tong University	China	44	2.23
Jilin University	China	40	2.03
Zhejiang University	China	34	1.73
Huazhong University of Science and Technology	China	31	1.57
University of Chinese Academy of Sciences	China	30	1.52
Nanjing University	China	28	1.42
Central South University	China	27	1.37

The University of California San Diego had the greatest contribution, publishing 93 articles concerning cell membrane‐coated nanoparticles (Figure 3D). Its leading scholar was Prof. Liangfang Zhang, who was the pioneer of cell membrane coating nanotechnology. The second productive institution was Wuhan University (*n* = 89), and the leading scholars were Wei Zhang, Wei Liu, Qianfang Meng, and Lang Rao. Their main research directions were the wound‐targeting function of platelet to achieve fixed‐point illumination of the wound and hybrid cell membrane (HM)‐coated nanoparticles for disease diagnosis and treatment.^[^ ^12^, ^13^ ^]^ The visualization of the collaborations among institutions is presented in Figure 3E, distributed in four clusters led by the University of California San Diego, Wuhan University, and the University of Chinese Academy of Sciences. Fudan University and Shanghai Jiao Tong University made basically the same contribution. The four clusters also internally communicated with each other. There was cooperation among the four clusters and small institutions, as well as some cooperation among small institutions. In conclusion, the collaborations among institutions were not gathered around, which meant that it still had room for improvement in future cooperation.

### 2.4 Co‐Occurrence Network Analysis of Keywords

**Figure 4:**
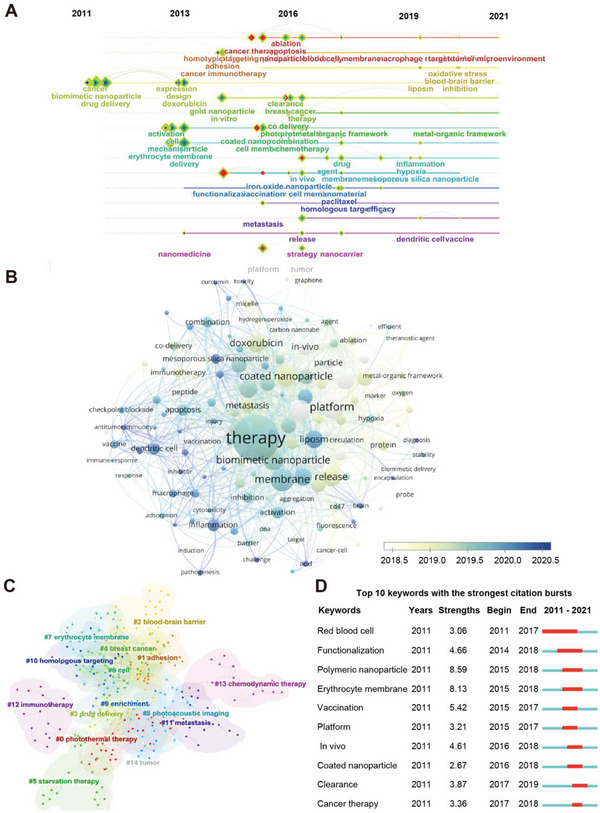
Keywords co‐occurrence network and citation bursts were applied in the field of cell membrane‐coated nanoparticles. A) The keywords timeline map from 2011 to 2021. B) Network visualization map of keywords started from 2018 to 2020. C) 15 keyword clusters were generated. D) The top ten keywords with the strongest citation bursts in this field (blue bubbles: most important keywords from 2011 to 2016; yellow bubbles: most important keywords from 2017 to 2021).

Citespace software was used to generate the cluster analysis and timeline of the keywords burst diagram (modularity *Q* = 0.7088, weighted mean silhouette *S* = 0.8601). As presented in **Figure** **4**A, from 2011 to 2016, the keywords mainly related to the disguise of cell membranes to nanoparticles which enhanced the circulation time in vivo and they acted as carriers to load chemo regiments; during the past 5 years, the keywords evolved to biomimetic and engineering nanoparticles, applying in different scenarios such as photodynamic therapy, checkpoint blockade, immunogenic cell‐death, and stem‐cell (Figure 4B). The hotspots of recent studies were tumor microenvironment, blood–brain barrier, oxidative stress, metal‐organic framework, inflammation, hypoxia, and dendritic cell vaccine, which encourage researchers to pay attention to. Eleven clusters were grasped based on the *K* values (g‐index *k* = 25, Figure 4C), starting with “adhesion,” “blood–brain barrier,” “drug delivery,” and so on. We continued to search out the top ten keywords with the strongest citation bursts (**Table** **4**). We could see “red blood cell” and “erythrocyte membrane” had a profound influence in this field (Figure 4D), as the use of RBCM camouflage is currently the most mature and convenient approach; later the strongest citation bursts altered to “functionalization,” “vaccination,” and “clearance.” It could be concluded that the core technology is to use different functions of cell membranes and nanomaterials to keep up with cutting‐edge research for suitable medical scenarios.

**Table 4:** The top ten frequent words concerning cell membrane‐coated nanoparticles

Rank	Words	Occurrences	Counts [%]
1	nanoparticle	192	4.77
2	delivery	156	3.87
3	drug‐delivery	113	2.81
4	therapy	84	2.09
5	cell	75	1.86
6	cancer	69	1.71
7	polymeric nanoparticle	53	1.32
8	platform	38	0.94
9	membrane	37	0.92
10	mechanism	36	0.89

### 2.5 Growing Productions in Journals and Collaborations

**Figure 5:**
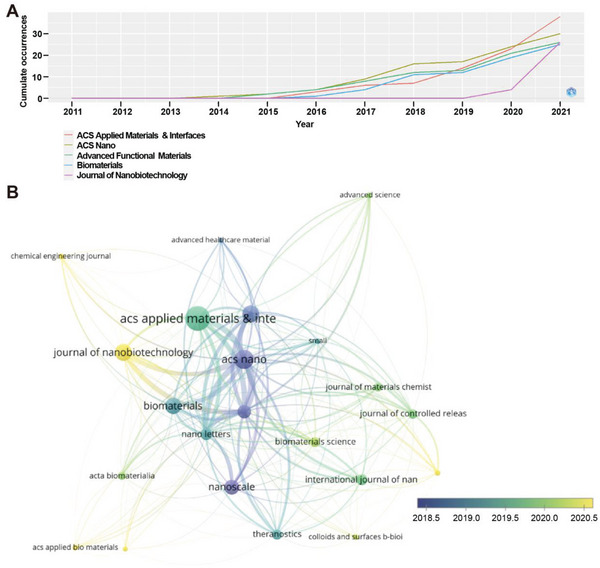
Co‐occurrence network of journals in the cell membrane‐coated nanoparticles field. A) The production trends of journals changed with time. B) Timeline‐based co‐occurrence network of journals (node sizes: publication numbers).

A total of 143 journals that published articles on the subject of cell membrane‐coated nanoparticles have been collected. The top ten ranking journals are listed in **Table** **5**, with ACS Applied Materials & Interfaces (*n* = 38) being the most productive journal in this field. It was followed by ACS Nano (*n* = 30), Advanced Functional Materials (*n* = 26), Journal of Nanobiotechnology (*n* = 26), and Biomaterials (*n* = 25). The above journals were of high quality in the field of biomedical science and material science. It revealed that cell membrane‐coated nanoparticles attracted academic attention and exerted a great scientific impact on scholars and their studies. From the developing trend in this field, ACS Nano first rose in 2014, publishing a work entitled “Erythrocyte membrane is an alternative coating to polyethylene glycol for prolonging the circulation lifetime of gold nanocages for photothermal therapy” (https://doi.org/10.1021/nn503779d).^[^ ^14^ ^]^ Next, it came with Advanced Functional Materials in 2015, ACS Applied Materials & Interfaces and Biomaterials in 2016, and Journal of Nanobiotechnology in 2020 (**Figure** **5**A).

**Table 5:** The top ten productive journals concerning cell membrane‐coated nanoparticles

Rank	Journals	Counts	Counts [%]
1	ACS Applied Materials & Interfaces	38	15.83
2	ACS Nano	30	12.50
3	Advanced Functional Materials	26	10.83
4	Journal of Nanobiotechnology	26	10.83
5	Biomaterials	25	10.42
6	Nanoscale	23	9.58
7	Advanced Materials	21	8.75
8	Nano Letters	18	7.50
9	International Journal of Nanomedicine	17	7.08
10	Theranostics	16	6.67

The journal‐citation network started with Nanoscale in 2018, then it continued in ACS Applied Materials & Interfaces, Biomaterials, and Nano Letters in 2019. In 2020, the collaborations mostly launched among Journal of Nanobiotechnology, ACS Applied Bio Materials, and Chemical Engineering Journal (Figure 5B). The results indicated that there were close academic citations between journals, and it was still growing.

From the aspect of journal type, it also indicated that related journals altered from nanomaterials‐related journals to comprehensive journals related to biology and chemistry. In order to straighten out relations among institutions, authors, and keywords, we conducted a three‐field plot by R bibliometrix package (Figure S1, Supporting Information). It suggested that research fields are overlapping, and institutional citations need to be strengthened.

### 2.6 Foam‐Tree Analysis of Hotspots in Cell Membrane‐Coated Nanoparticles

**Figure 6:**
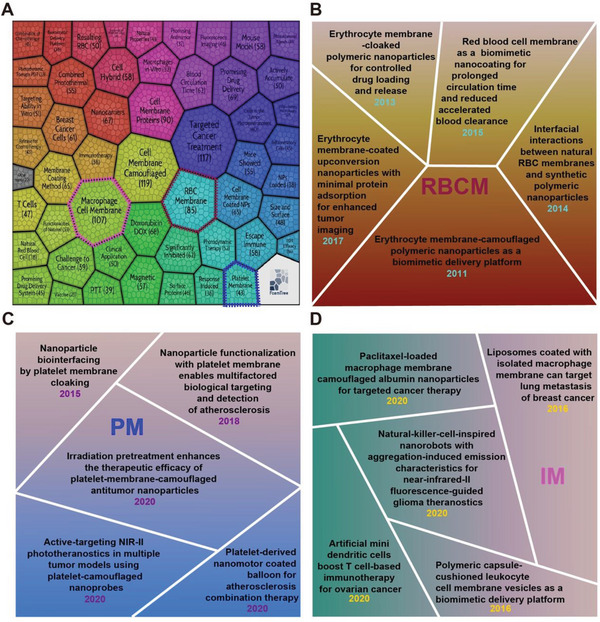
Foam‐tree analysis in the cell membrane‐coated nanoparticles field. A) Research hotspots with different topics were presented in various clusters. B) Representative works in RBCM, PM (C), and IM (D).

To decipher the frontier hotspots of cell membrane‐coated nanoparticles field more adequately, we performed hotspot foam‐tree analysis via a website‐based analyzing tool—Carrot^2^. Each foam represented a certain field, and the size of the foam represented the number of papers (**Figure** **6**A). The results demonstrated that the most influential hotspots were “Cell membrane camouflaged,” “Targeted cancer treatment,” “Macrophage cell membrane,” “Cell membrane proteins,” and “RBC membrane.” Among these clusters, we could find that different cell membranes were gathered into independent clusters, such as “RBC membrane (*n* = 119),” “Macrophage cell membrane (*n* = 107),” “Platelet membrane (*n* = 43),” “T cell (*n* = 47),” and “Breast cancer cells (*n* = 61).” To further understand the specific medical application scenarios of each cell membrane, we enumerated the research related to RBCM in Figure 6B, PM in Figure 6C, and IM in Figure 6D. It reflected that current research trends still on different biomedical applications after materials were coated with different cell membranes.

### 2.7 Separated Analysis of Different Cell Membrane‐Coated Nanoparticles and Perspectives of Research Frontiers

**Figure 7:**
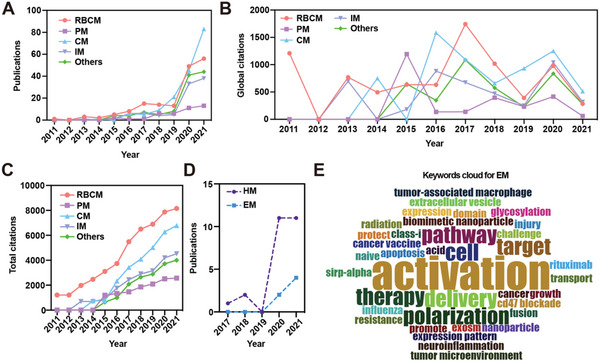
Application summaries of different cell membranes in the cell membrane‐coated nanoparticles field. A) The number of publications in five types of cell membranes; the global citations each year and the total citations are shown in (B) and (C). D) The number of publications in HM and EM; the keywords cloud for EM is generated in (E).

To properly facilitate researchers to choose the suitable cell membrane for their research direction, we continued to explore how medical applications of different cell membrane‐coated nanoparticles varied in the number of publications, citations, and topic trends. After categorizing the cell membranes into five types, namely RBCM, PM, CM, IM, and others, respectively, we found that RBCM was with the greatest number of publications from 2011 to 2018 (total *n* = 166, **Figure** **7**A); the CM ushered in explosive growth in 2019 and became most popular in 2021 (total *n* = 169); IM had slow growth from 2016 to 2019, followed by moderate growth after 2019 (total *n* = 94); PM was more niche than other types (total *n* = 40). Although it started early, the research has grown rather slowly. Others included HM, stem cell membranes, and cardiomyocytes (total *n* = 113). It was worth mentioning that publications in others were growing fast since 2019. It indicated that the applications of cell membrane‐coated nanoparticles have expanded to broader medical treatment. In the aspect of global citations, RBCM had the most cited work in 2011 (*n* = 1209, Figure 7B), then it was the most cited again in 2017 (*n* = 1743); PM reached its citation peak in 2015 (*n* = 1194), while IM had it in 2020 (*n* = 1041); the popularity of CM had not diminished since 2014, and there have been three peaks in citations in 2014 (*n* = 751), 2016 (*n* = 1584), and 2020 (*n* = 1250); while others had a trend of becoming popular on citations since 2015.

For an in‐depth understanding of the result, separate analyses were performed. As for the development of RBCM, the most productive country was China (Figure S2A, Supporting Information), followed by the USA and Finland. The global article productivity of RBCM‐coated nanoparticles is shown in Figure S2B, Supporting Information. ACS Nano, ACS Applied Materials & Interfaces, and Biomaterials were the top three most relevant sources in this field, with Fudan University being the most productive institution (Figure S2C,D, Supporting Information). Liangfang Zhang and his team members had the highest output in this field from 2011 to 2021 (Figure S2E, Supporting Information). There were mainly six clusters concerning the cooperation among authors. An in‐depth analysis revealed that the cooperation stayed within the authors’ own institutions other than cooperation among institutions, which indicated that it should be strengthened in the future (Figure S2F, Supporting Information). As shown in Figure S2G, Supporting Information, the 5‐year topic trends in RBCM started with “erythrocyte membrane,” and “polymeric nanoparticle” to “delivery” and “nanoparticle.”

China, the USA, and South Korea were the top three most productive countries in the development of PM (Figure S3A, Supporting Information). Figure S3B, Supporting Information, depicts the global PM‐coated nanoparticle article productivity. The top three most relevant sources included ACS Applied Materials & Interfaces, Advanced Materials, and ACS Nano, with the University of California San Diego being the most productive institution (Figure S3C,D, Supporting Information). In the span of 7 years, Liangfang Zhang and his team members produced the most (Figure S3E, Supporting Information). Seven clusters were generated in terms of collaboration among authors. Liangfang Zhang, Ronnie H Fang, and Weiwei Gao were located at the center of the collaboration (Figure S3F, Supporting Information). The terms “polymeric nanoparticle” and “drug delivery” to the term “nanocarrier” marked the beginning of the 5‐year topic trends in PM (Figure S3G, Supporting Information).

As for the tendency of CM development, China, the USA, and Italy were the top three most productive countries (Figure S4A, Supporting Information). Articles about CM‐coated nanoparticles globally are shown in Figure S4B, Supporting Information. ACS Applied Materials & Interfaces was the most relevant journal in the CM field, followed by Biomaterials, and Nano Letters. Wuhan University was the most productive institution (Figures S4C,D, Supporting Information). In this emerging field, Xianzheng Zhang, Jun Feng, and Hong Cheng from Wuhan University paid more attention (Figure S4E, Supporting Information). Nine clusters were generated in terms of collaboration among authors. It suggested that CM‐coated nanoparticles might have a broad medical application prospect, as many authors and institutes had the interest to launch research focusing on CM (Figure S4F, Supporting Information). The 5‐year topic trends in CM started with “immunotherapy,” “drug delivery” to “breast cancer,” and “expression,” indicating that CM was carried out to confer the immune activation ability of nanoparticles (Figure S4G, Supporting Information).

In terms of the analysis of IM, China, the USA, and Italy also rounded out the top three most productive nations (Figure S5A, Supporting Information). The global production presented that articles in this field were mainly in East and West Asia and North America (Figure S5B, Supporting Information). The top three most relevant journals included Journal of Nanobiotechnology, ACS Nano, and Biomaterials. Additionally, Wuhan University was also the most productive institution (Figure S5C,D, Supporting Information). In this topic, Qiang He from the Harbin Institute of Technology and Junbai Li from the Chinese Academy of Sciences had multi‐year sustained output (Figure S5E, Supporting Information). Nine clusters were generated in terms of collaboration among authors, with Lintao Cai, Liangfang Zhang, and Meiyu Song being the leading positions (Figure S5F, Supporting Information). Figure S5G, Supporting Information, depicts the 5‐year topic trends in IM, which included “cancer” and “delivery” to “platform” and “in vivo.” It could be concluded that the functions of the cell membranes were well‐clarified, and the hotspots had altered to use them as nanocarriers to enhance the delivery of nanoparticles.

To further analyze the influence of different cell membranes in the field, we added up the citations of each type of membrane each year to observe the overall citation changes. As presented in Figure 7C, the total citations of PM and IM increased relatively slowly compared with RBCM and CM; the emergence of others represented new blood in this field, as it accelerated fast since 2014. HM arouse when multiple functions were desired to occur on the cell membranes. Considering that many strategies were too complex for clinical translation, the concept of EM began to emerge. To achieve the goal of maintaining antigenicity on CM while achieving long circulation, the HM of the CM and RBCM appeared. Zhang et al. designed the engineered CM in 2021, which could overexpress CD80 to directly stimulate T cells to respond. It avoided complex antigen presentation reactions, and directly changed the tumor microenvironment from “cold” to “hot,” achieving the effect of serving multiple purposes.^[^ ^15^ ^]^ Furthermore, we selected HM and engineering cell membranes (EM) from others to see the developing trend. Figure 7D shows that both HM and EM were novel topics and had satisfied growth rates.

Concerning the analysis of other cell membrane‐coated nanoparticles, China, the USA, and France were the top three most productive countries (Figure S6A, Supporting Information). Articles in this field were dispersed throughout Asia and the United States as a result of global production (Figure S6B, Supporting Information). Nanoscale, Advanced Materials, and ACS Applied Materials & Interfaces were the top three most relevant journals. In this field, the most productive institution was the University of California San Diego (Figure S6C,D, Supporting Information). The members of Liangfang Zhang's team produced the most since 2015 (Figure S6E, Supporting Information). Seven clusters were produced in terms of collaboration among authors, with Liangfang Zhang and his team membranes deeply involved in this field (Figure S6F, Supporting Information). The 5‐year topic trends in others started with “strategy,” “erythrocyte membrane,” then moved to “nanoparticles” and “delivery” (Figure S6G, Supporting Information). Since EM could reflect the advantages of different cell membrane components, it had great potential in future research. We grasped the keywords cloud for EM in Figure 7E. It indicated that underlying biological mechanisms based on the treatment of cell membrane‐coated nanoparticles aroused great interest in the perspective of this field.

## 3 Discussion

In this study, we analyzed the main developmental knowledge and trends of cell membrane‐coated nanoparticles in the biomedical field. Since the emergence of RBCM‐coated nanoparticles, different cell membranes, such as PM, CM, IM, and others, were applied in various medical scenarios. China was the most productive country among 31 countries (Figure 1E). The University of California San Diego was the most productive institution, which was located in the USA, followed by Wuhan University which was located in China (Figure 3D). Liangfang Zhang was the most representative scholar in this field with the most cited article (Figure 3A),^[^ ^2^ ^]^ and his team mainly focused on solving the stability and targeted drug delivery of nanoparticles in vivo and carrying out clinical translation.^[^ ^3^, ^12^, ^16^ ^]^ Their latest work is on mimicking virus‐like membrane‐coated nanoparticles to deliver mRNA, using viruses’ nature of triggering membrane fusion and thereby delivering mRNA cargoes intracellularly.^[^ ^17^ ^]^

Despite various cell sources for cell membranes, the extraction and purification process, as well as the coating methods are quite similar (Scheme 1). Moreover, the physical and biological characteristics of cell membranes, such as size, specific protein expression, and zeta potential, are also alike. For non‐nuclei cells, cell membranes are easier to obtain and widely used. It mainly includes erythrocytes and platelets. As shown in Figure 4C, erythrocyte membrane ranked 8th in keyword searching, which confirms its widespread use. Coating RBCMs can help to avoid identification by macrophages for they express CD47, a protein that assists immune escape, resulting in longer circulation time for nanoparticles in vivo.^[^ ^18^ ^]^ Platelet encapsulation also helps evade the clearance of the immune system, promoting circulation time, and enhancing the drug concentration in the focus. Furthermore, Jiang et al. reported that platelet membranes‐coated magnetic nanoparticles can repolarize immunosuppressive M2 macrophages into anti‐tumor M1 phenotype.^[^ ^19^ ^]^ Moreover, a PM‐coated biomimetic nanocarrier has been proven to possess functions of not only decreasing off‐target effect at the tumor site but also thrombosing to cut off nutrition for tumors.^[^ ^20^, ^21^ ^]^ Cancer treatment is a major problem that mankind is currently working hard on. It can be seen that in Figure 4B,C, the cancer‐related topics of membrane‐coated nanoparticles include “breast cancer,” “metastasis,” “immunotherapy,” “chemodynamic therapy,” and “tumor.” CM has the unreplaceable advantage of serving as the nano‐tumor vaccine that stimulates the body's humoral or cellular immunity more effectively to tumor cells in comparison with traditional anti‐cancer therapies.^[^ ^22^ ^]^ A major dilemma of current tumor therapy is that tumor cell immune escape leads to insufficient efficiency of antigen‐presenting cells (APC) presentation and the low specificity of the extracted tumor antigens, coating CM perfectly addressing the dilemma through targeting APCs, promoting homologous recognition between materials and tumor cells, and precise targeting of tumor cells. Gou et al. reported that engineered peptide‐expressed biomimetic CM‐encapsulated STING agonists can significantly enhance type I interferon (IFN) secretion in dendritic cells and inhibit PD‐1 unresponsive tumor growth without causing obvious side effects.^[^ ^23^ ^]^ Furthermore, Wang et al. camouflaged Ce6 with CM to perform a combination of photodynamic therapy and hyperthermia for tumor remote metastasis, which resulted in a synergistic effect in tumor inhibition by triggering immunogenic cell death.^[^ ^24^ ^]^

As for IM, APCs represented by macrophages, natural killer cells (NKs), and cytotoxic lymphocytes (CTLs) all belong to the category of immune cells. The mechanism of macrophage membranes to fight tumors is based on the recognition between integrin α4 and vascular cell adhesion molecule (VCAM) expressed on the surface of CM.^[^ ^25^, ^26^ ^]^ pH‐sensitive nanomaterials coated by the macrophage membranes can be stably released in the blood circulation (pH 6.5), but the rapid release occurs after membrane fusing in the intratumoral acid microenvironment (pH 5), which helps to precisely kill tumor cells.^[^ ^27^ ^]^ Studies on NK cell membrane‐encapsulated nanoparticles have exhibited that this biomimetic strategy can utilize receptor molecules on the surface of NK cell membranes. This camouflage not only prolongs the circulation time in vivo but also excites significant tumor‐homing potential, which is expected to play a role in tumor bioimaging and immune monitoring.^[^ ^28^ ^]^ As the specific immune recognition receptors are expressed on the surface, T cell membrane coating is promising.^[^ ^29^ ^]^ Zhang et al. designed nanoengineered CD4+ T cell membrane‐coated nanoparticles to integrate with HIV‐1 strains and to target gp120 high‐expressing cells to reduce HIV sanctuaries.^[^ ^30^ ^]^ Other membranes include stem cell membranes, out membrane vesicles (OMVs), endothelial cell membranes, islet beta cell membranes, and hybrid membranes. Stem cell membranes tend to have low immunogenicity and possess the ability to repair and regenerate, which decreases the toxicity of nanoparticles in the lung and rescues damaged myocardium by reducing apoptosis.^[^ ^9^, ^31^ ^]^ On the opposite, OMVs have strong immunogenicity and are suitable for the preparation of antibacterial vaccines.^[^ ^32^ ^]^ It activates dendritic cells in the lymph nodes in vivo and promotes the maturation of DCs, triggering durable antibody responses, inducing the production of IFN‐γ and IL‐17, and stimulating T cells to respond.^[^ ^33^ ^]^ Islet beta cell membranes can utilize the interaction between β cells to promote the growth and reproduction of residual β cells, to provide therapeutic ideas for effectively solving insufficient insulin production.^[^ ^34^ ^]^ Due to the affinity and adhesion between endothelial cells and innate immune cells, wrapping the endothelial cell membranes around anti‐infective drugs can effectively deliver drugs to the site of inflammation.^[^ ^8^ ^]^

Through our analysis, we found that HM and EM have the therapeutic effect of fusion and multi‐function, which is one of the main development directions of cell membrane‐encapsulated nanomaterials in the future (Figure 7D).^[^ ^35^ ^]^ HM not only significantly improves the specific recognition of nanoparticles to certain cell lines and accelerates the intra‐cellularization but also extends the blood retention time of nanoparticles. The circulation lifetime of HM‐encapsulated nanoparticles was 1.39 times and 3.88 times longer than CM‐encapsulated nanoparticles and pure nanoparticles after 24‐h injection.^[^ ^36^ ^]^ Hybrid biomimetic coating of chemotherapy regimen doxorubicin and photosensitizer CuS exhibits great synergistic photothermal/chemotherapy with ≈100% inhibition of melanoma growth.^[^ ^36^ ^]^ Combinations of CM and OMV achieve a better clinical therapeutic effect by enhancing the innate immune response and residing in lymph nodes, reducing the risk of metastasis.^[^ ^37^ ^]^ Cell membrane surface engineering can serve different purposes. One of its most prominent applications is the introduction of targeting moieties (Figure 7E). By providing cell membranes with these fractions, their selective enrichment in specific cells and underlying tissues can be achieved. Take vaccines for example, surface modification of EM can both help vaccines maintain antigenicity and improve safety in the meantime.^[^ ^38^ ^]^ Genetic engineering strategies for EM surface functionalization are also based on the transgenic expression of proteins or chimeric proteins. OMVs of transgenic pDNA‐TRAIL (a pro‐apoptotic gene) *Escherichia coli* modified with an integrin αvβ3 peptide targeting ligand and indocyanine green (designated IP‐OMVs) acted as combo punch to treat cutaneous melanoma.^[^ ^39^ ^]^ With the assistance of near‐infrared irritation, it can not only arouse photothermal–photodynamic responses against primary melanoma but also activates TRAIL‐related apoptosis in disseminated tumor cells, leading to a complete eradication of the tumor. At present, cell membrane‐encapsulated nanoparticles are also gradually transforming into clinical applications, among which erythrocyte membranes are the most widely used strategies for regimen encapsulation (Table S1, Supporting Information). Erythrocytes encapsulating l‐asparaginase (GRASPA) have launched phase II/III trials in acute lymphoblastic leukemia and acute myeloid leukemia (NCT01518517 and NCT01810705). Ery‐Dex (dexamethasone sodium phosphate encapsulated in autologous erythrocytes) was also applied in nervous system disorders and genetic syndrome (NCT01255358 and NCT02380924). Apart from the treatment of erythrocyte membranes, debridement + NanoPRP coverage treatment based on PM is currently recruiting patients with diabetic foot ulcers (ChiCTR2100046769). Preclinical trials, including delivery of immunotherapy agents and delivery of chemotherapy agents, are ongoing (CE119, PNP‐R848, CE118, etc). Clinical trials of cell membrane‐coated therapeutics are emerging, indicating their relatively promising medical translational prospects.

According to different diseases or research purposes, choosing different membranes to fuse or to modify membrane surface proteins through gene editing can exert a variety of cell membrane functions, which is an important development direction in this field.

## 4 Conclusions

For better diagnosis approaches, disease prevention, and treatment, cell membrane‐encapsulated nanoparticles have broad prospects in clinical translation. Bio‐inspired nanotherapeutic strategies provide an innovative platform for this purpose. Drug delivery, detoxification, and immunomodulation are the three main research directions for current medical applications of cell membrane‐encapsulated nanoparticles. We identified historical and future research trends by analyzing the number of publications and citations, the productive countries, influential and high‐cited authors and institutions, mainstream journals, representative works, co‐occurrence keywords, and frontier hotspots in different cell membranes during the past 11 years of expeditious development in this field. As noted in the result section, institutions from the China and USA play leading roles with other countries and institutions steadily participating. From the trends concerning global cooperation, it becomes more and more tight and prosperous as time goes by. Moreover, Liangfang Zhang and his team contributed the most in this field, with the University of California San Diego being the institution with the highest output. Recent researches pay more attention to the applications of HM and EM‐encapsulated versatile nanoparticles in complicated medical scenarios by taking advantage of the specific underlying mechanism of molecules on the membrane. Different from meta‐analysis and general review, bibliometric analysis is the basis of our study, which offers a more comprehensive and unique aspect of the growth of cell membrane‐coated nanoparticles. Given that this field is gradually becoming marketable, we are looking forward to better and more cutting‐edge research.

## 5 Experimental Section

### Data Sources and Search Strategies

Data were collected from WoSCC with the algorithm of TS = (nanoparticles*) AND ( ( ( (TS = (coat*)) OR TS = (camouflage*)) OR TS = (biomimetic)) AND TS = (cell membrane)) on July 26th, 2022. The literature research schedule ranged from January 1st, 2011 to December 31st, 2021. A total of 3478 publications were retrieved, while 390 articles, including reviews (*n* = 322), meeting articles (*n* = 71), meeting abstracts (*n* = 16), book chapters (*n* = 6), editorial material (*n* = 4), revisions (*n* = 2), online publications (*n* = 2), retraction (*n* = 2), data papers (*n* = 1), reprint (*n* = 1), retract content (*n* = 1), and non‐English (*n* = 16) were removed. Finally, the title, abstract, or full text of these publications were read to screen out articles closely related to the topic studied (cell membrane‐coated nanoparticles), and the criteria for the collected data included article type, publication year, institution, country, authorship, abstract, keyword, citation, and references. After careful selection, a total of 583 publications were retrieved and analyzed. The publication list of the 583 articles is provided in Table S1, Supporting Information.

### Data Analysis

CiteSpace.6.1.R3 and 5.8.R1, R bibliometrix package V 3.2.1, Carrot^2^ workbench 3.10.2, and VOSviewer 1.6.18 were applied in this bibliometric analysis. Citespace was used to perform cluster analysis containing references, keywords, and timeline views.^[^ ^40^ ^]^ A modularity *Q* > 0.3 and a mean silhouette > 0.5 were considered qualified for the clustering results. Citespace was used to illustrate the top six productive countries, and *R* was used to show the top ten influential authors.^[^ ^41^ ^]^ The collaborative connections between countries, institutions, authors, and journals were also produced by R. R bibliometrix package was used to conduct three‐field plots and the bibliometric geographic map.^[^ ^42^ ^]^ Moreover, the keyword density maps of cell membrane‐coated nanoparticles field worldwide were generated by Vosviewer. Graphs of publications and citations presented in this study were conducted by GraphPad Prism 8.3.0.

## Conflict of Interest

The authors declare no conflict of interest.

## Author Contributions

Y.Z. and S.J. contributed equally to this work. Conceptualization: Y.Z., S.J., X.F., Q.M.; Methodology: S.J., Y.Z., G.C., Y.C.; Investigation: Q.X., S.J., D.L., Y.L.; Visualization: S.J., Y.Z.; Supervision: H.L.; Writing–original draft: Y.Z., S.J.; Writing–review & editing: Y.Z., J.Y., X.F., H.L.

## Supporting information

Supporting Information

## Data Availability

The data that support the findings of this study are available from the corresponding author upon reasonable request.
