# Supplementary material for: A Machine‐Learning‐Based Bibliometric Analysis of Cell Membrane‐Coated Nanoparticles in Biomedical Applications over the Past Eleven Years
Source: Glob Chall. 2023 Feb 22;7(4):2200206. doi: 10.1002/gch2.202200206 (PMC10069317; doi:10.1002/gch2.202200206)
Supplement: Supplementary file 1 — Supporting Information [file GCH2-7-2200206-s001.pdf]

Global Challenges
Open Access

Supporting Information

for *Global Challenges,* DOI: 10.1002/gch2.202200206

A Machine-Learning-Based Bibliometric Analysis of Cell Membrane-Coated Nanoparticles in Biomedical Applications over the Past Eleven Years

Yiyin Zhang, Shengxi Jin, Duguang Li, Guoqiao Chen, Yongle Chen, Qiming Xia, Qijiang Mao, Yiling Li, Jing Yang, Xiaoxiao Fan, and Hui Lin**

Supporting Information

A Machine-Learning-Based Bibliometric Analysis of Cell Membrane-Coated Nanoparticles in Biomedical Applications over the Past Eleven Years

Yiyin Zhang[#], Shengxi Jin[#], Duguang Li, Guoqiao Chen, Yongle Chen, Qiming Xia, Qijiang Mao, Yiling Li, Jing Yang, Xiaoxiao Fan[*], Hui Lin[*]

Y Zhang, S Jin, D Li, G Chen, Y Chen, Q Xia, Q Mao, Y Li, J Yang, X Fan, H Lin
Department of General Surgery, Sir Run Run Shaw Hospital, School of Medicine, Zhejiang University, Hangzhou, 310016, China.
E-mail: 369369@zju.edu.cn (H.L.), fanxx_gs@zju.edu.cn (X.F.)

H Lin
Zhejiang Engineering Research Center of Cognitive Healthcare, Sir Run Run Shaw Hospital, School of Medicine, Zhejiang University, 310016, China.
E-mail: 369369@zju.edu.cn (H.L.)

[#]*These authors contributed equally to this work.*

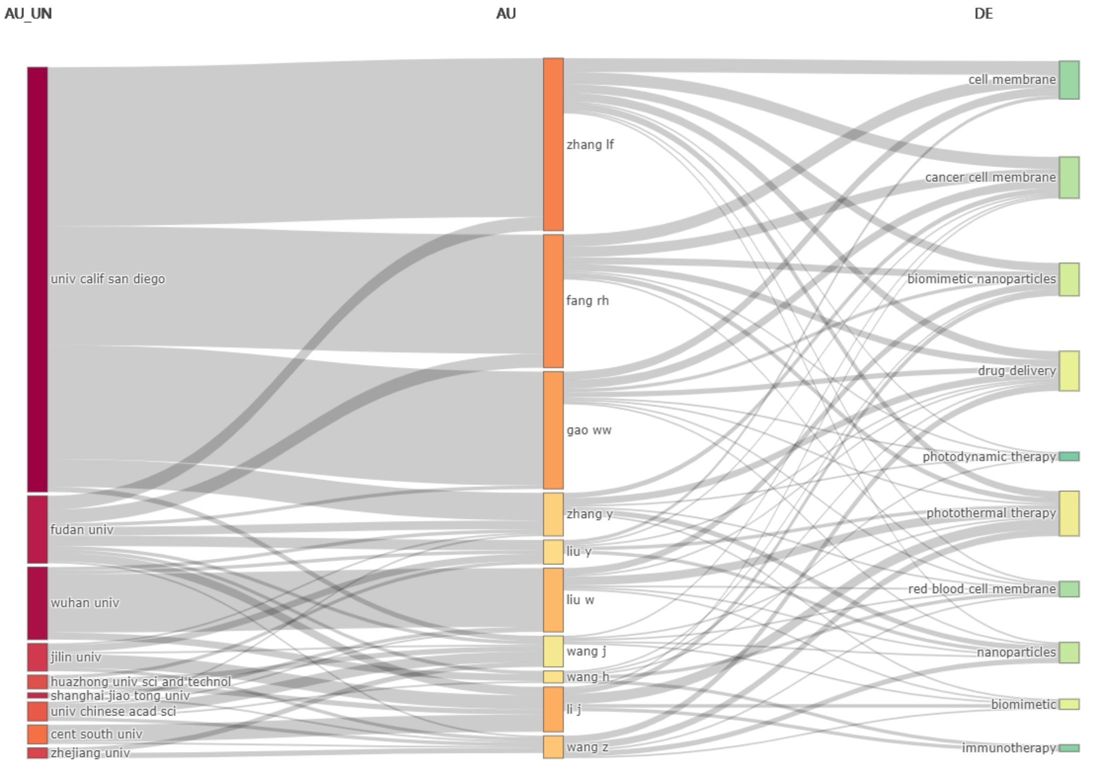

Figure S1. Three-fields plot of institutions, scholars, and related keywords in this field.

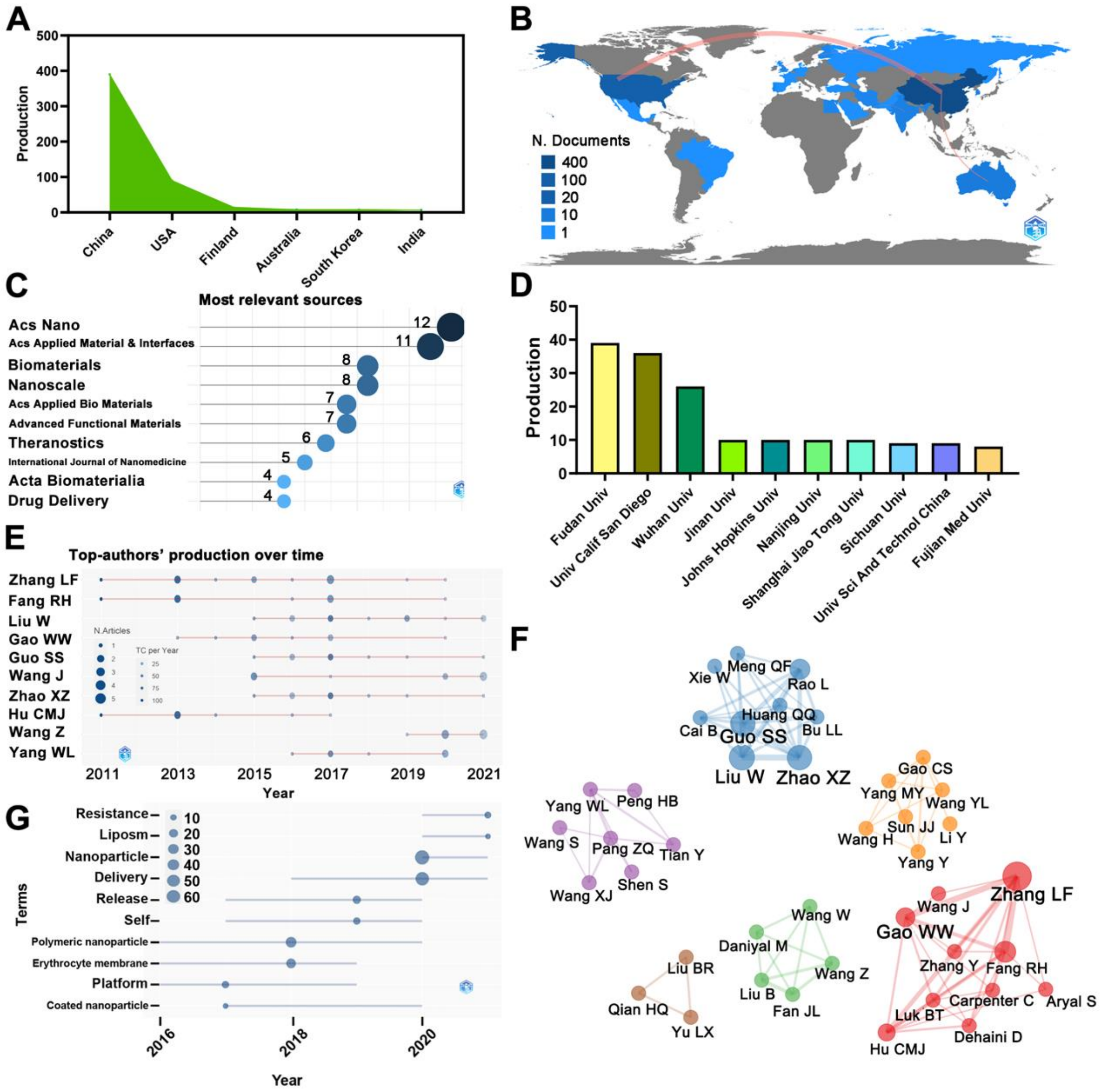

Figure S2. Bibliometric analysis of RBCM-coated nanoparticles research. (A) The number of publications in the top 6 countries; (B) the global production was shown. (C) The top 10 most relevant sources. (D) The top 10 most productive institutes, the top 10 most productive authors (E), and collaboration among authors (F). (G) A 5-year trend topics analysis.

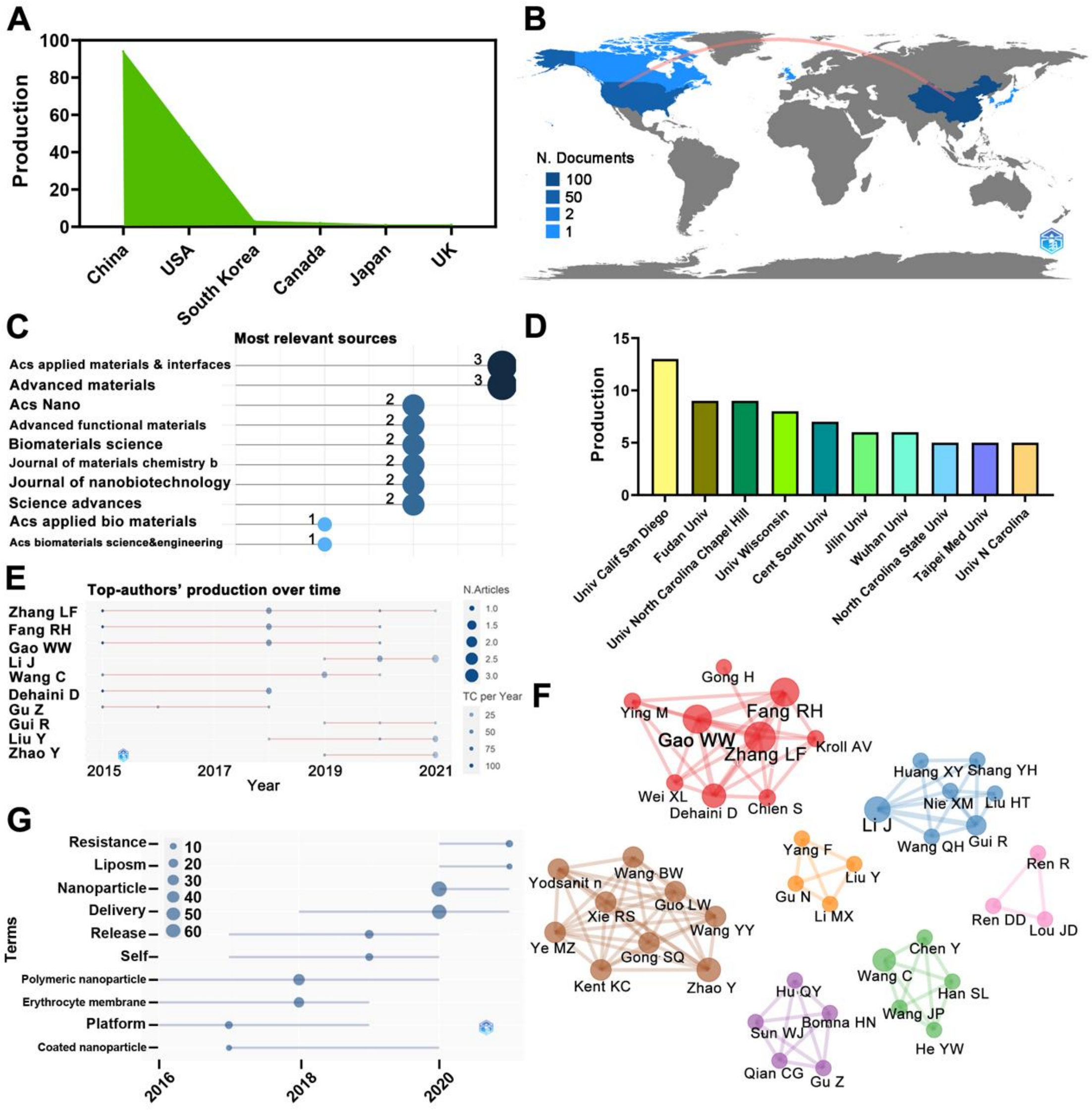

Figure S3. Bibliometric analysis of research in PM-coated nanoparticles. (A) The top 6 countries contributed to the number of publications; the global production was shown in (B). (C) The top 10 most crucial sources. (D) The top 10 most productive institutes, the top 10 most influential authors (E), and the cooperative network among authors (F). (G) A 5-year trend topics analysis.

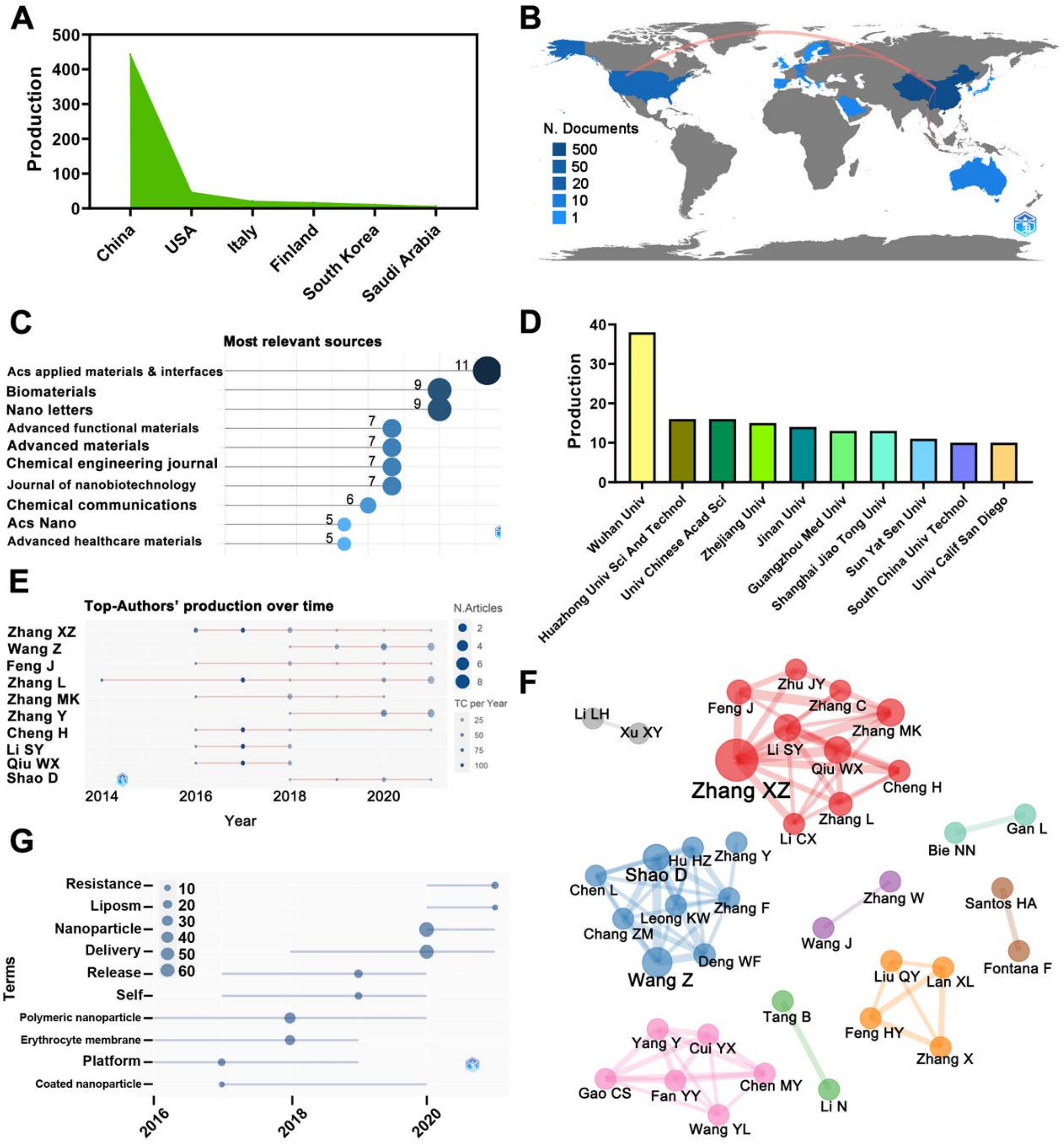

Figure S4. Bibliometric analysis of CM-coated nanoparticles research. (A) The number of publications in the top 6 countries; the global production was presented in (B). (C) The top 10 most relevant journals. (D) The top 10 most productive institutes, the top 10 most productive authors (E), and the network of author-to-author collaboration (F). (G) A trend topics analysis over five years.

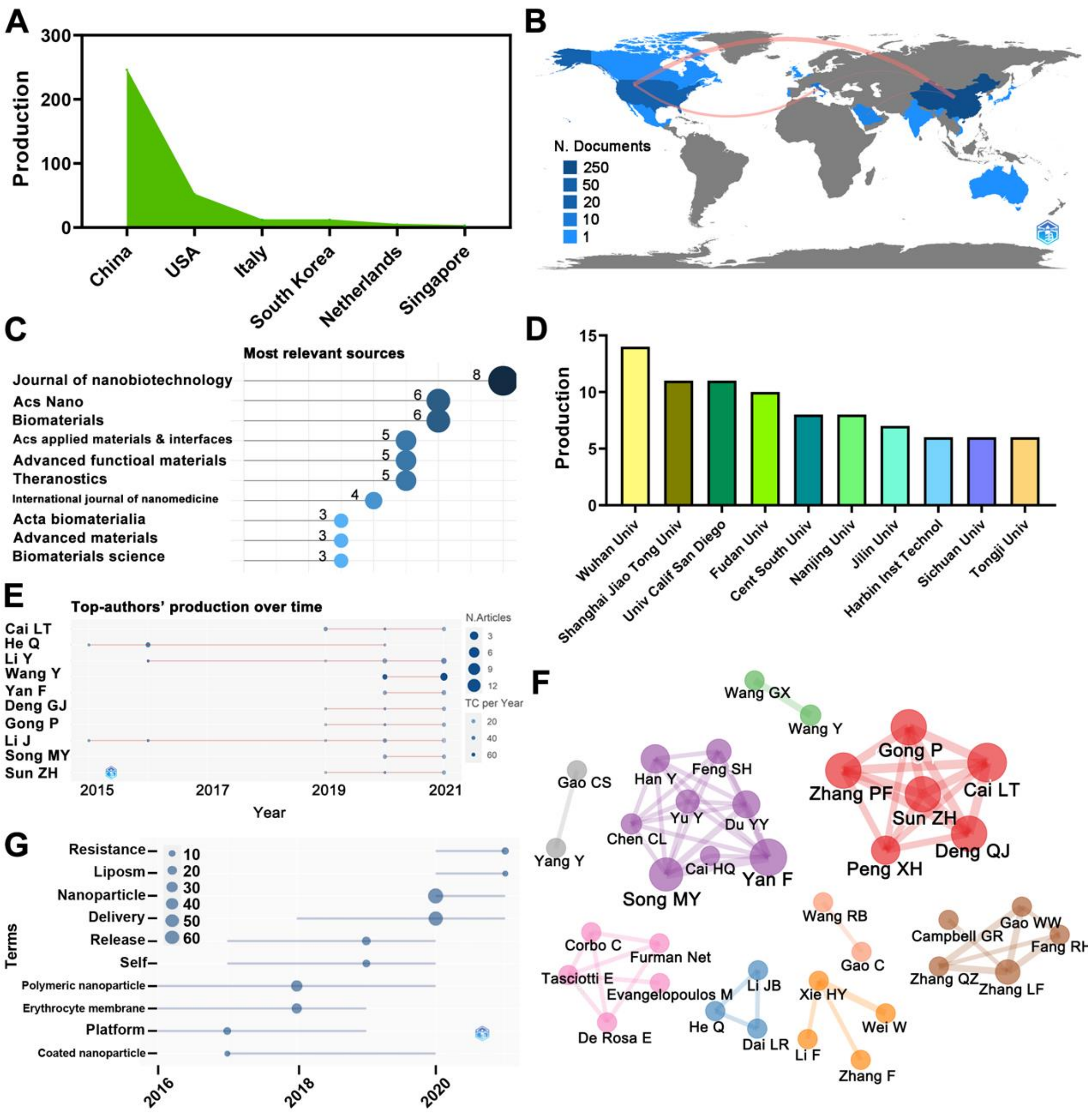

Figure S5. Bibliometric analysis of IM-coated nanoparticles research. (A) The number of publications in the top 6 countries. (B) The global production in this field. (C) The top 10 most constructive sources. (D) The top 10 most productive institutes, the top 10 most productive authors (E), and collaboration among authors (F). (G) A trend analysis of significant topics over 5 years.

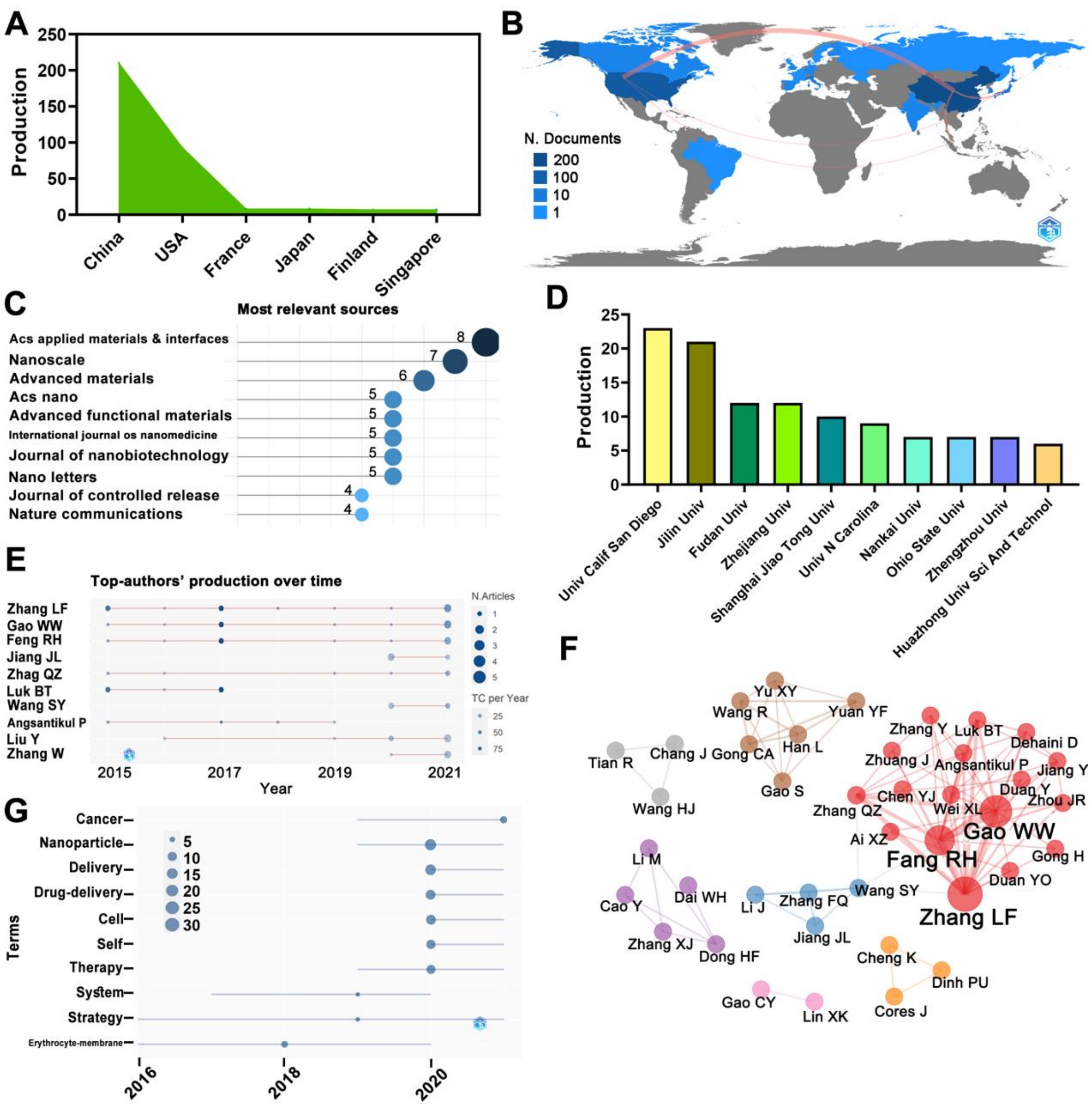

Figure S6. Bibliometric analysis of other cell membrane-coated nanoparticles research. (A) The number of publications in the top 6 countries; the research production globally was presented in (B). (C) The top 10 most relevant journals. (D) The top 10 most educational institutes, the top 10 most influential authors (E), and collaboration among authors (F). (G) A 5-year trend topics analysis.

Table S1. The ongoing clinical trials in the field of cell membrane-coated nanoparticles.

Category	Clinical Trial.gov ID	Institution	Disease	Study phase	Status	Treatment setting	Outcomes
Erythrocyte-membrane encapsulation	NCT01518517	Hopital Des Enfants Reine Fabiola, Hopital Saint Jacques, Hopital Pellegrin Enfants, etc	Acute Lymphoblastic Leukemia, in Relapse	II/III	Completed	Drug: Erythrocytes encapsulating L-asparaginase (GRASPA)	Duration of asparaginase activity >100 U/L during induction; allergic reaction during induction phase
	NCT03267030	Aahus University hospial, Tallin Childrens Hospital, Kuopio University Hospital, etc	Acute Lymphoblastic Leukemia	II	Completed	Drug: Eryaspase (asparaginase encapsulated in erythrocytes)	Pharmacokinetic parameters of 6 months (Enzyme activity (IU/L) and T1/2 (half-life time); area under the plasma concentration versus time curve (AUC); distribution Volume at steady state, etc Pharmacodynamic profile (Plasma concentrations of amino acids: asparagine, aspartate, glutamine, glutamate); Immunogenicity

						(Titers of anti-asparaginase antibodies and neutralizing antibodies)
NCT01925859	SNBL Clinical Pharmacology Center	Healthy	I	Completed	Drug: EryDex (dexamethasone sodium phosphate encapsulated erythrocytes)	Pharmacokinetic comparison of two different doses of EryDex in two independent groups
NCT02195180	Saint Catherine Institute, Institut de Cancerologie, Hopital Beaujon, etc	Pancreatic Adenocarcinoma Metastatic	II	Completed	Drug: ERY001 (L-asparaginase encapsulated in erythrocytes)	Overall survival (OS); Progression free survival (PFS)
NCT01523808	-	Pancreatic Cancer	I	Completed	Drug: GRASPA (Erythrocytes encapsulating L-Asparaginase)	Number of patients with dose-limiting toxicities up to week 4 after treatment
NCT01810705	Hôpital l'Archet 1, Institut Paoli Calmettes, Hôpital JEAN MINJOZ	Acute Myeloid Leukemia	II	Completed	Drug: GRASPA (Erythrocytes encapsulating L-Asparaginase)	OS

		, etc					
	NCT01523782	-	Acute Lymphoblastic Leukemia	II	Completed	Drug: GRASPA (Erythrocytes encapsulating L-Asparaginase)	Percentage of patients responding to treatment; dose limited toxicities assessed during induction 1 and induction 2
	NCT02380924	Dartmouth-Hitchcock Medical Center	Healthy	I	Completed	Drug: dexamethasone sodium phosphate loaded erythrocytes using EryDex System	24-hour post transfusion recovery of infused autologous erythrocytes
	NCT01255358	Spedali Civili and University La Sapienza	Nervous System Disorder; Genetic Syndrome	II	Completed	Drug: Ery-Dex (dexamethasone sodium phosphate encapsulated in autologous erythrocytes)	Changes in neurological symptoms assessed by using ICARS score
Platelet-membrane encapsulation	ChiCTR2100046769	Sir Run Run Shaw Hospital, Medical College of Zhejiang University	Diabetic Foot Ulcer	0	Recruiting	Debridement + NanoPRP coverage treatment	Wound area; RNA seq; immunofluorescence
	NCT04761562	University	Otitis Media	II/III	Recruiti	Tympanic Membran	Change of tympanic

		Medical Centre Ljubljana	Chronic		ng	e Perforation	membrane perforation size; change in chronic otitis media questionnaire 12 score
	NCT04281901	University Medical Centre Ljubljana	Otitis Media Chronic	-	Completed	Drug: Platelet- and extracellular vesicle-rich plasma ear wick soaked in platelet - and extracellular vesicle-rich plasma	Change in inflammation surface area; change in chronic otitis media questionnaire 12 score
	NCT04849429	Anupam Hospital	Chronic Low Back Pain	I	Completed	Biological: Platelet rich plasma (PRP) with exosomes	Visual analog scale; roland morris disability questionnaire; functional rating index, etc
Immune cell-membrane encapsulation	NCT04387071	HonorHealth Research Institute, USC/Norris Comprehensive Cancer Center, Hoag Memorial Hospital, etc	Locally Advanced Malignant Solid Neoplasm	I/II	Recruiting	Drug: VLP-encapsulated TLR9 Agonist CMP-001, and VLPs are detected and processed by cells of the immune system	Disease control rate (Phase II); Objective response rate (complete response rate + partial response rate)
